# Dietary Fiber from Chickpea (*Cicer arietinum*) and Soybean (*Glycine max*) Husk Byproducts as Baking Additives: Functional and Nutritional Properties

**DOI:** 10.3390/molecules24050991

**Published:** 2019-03-12

**Authors:** Guillermo Niño-Medina, Dolores Muy-Rangel, Ana Laura de la Garza, Werner Rubio-Carrasco, Briceida Pérez-Meza, Ana P. Araujo-Chapa, Kelsy A. Gutiérrez-Álvarez, Vania Urías-Orona

**Affiliations:** 1 Laboratorio de Química y Bioquímica, Facultad de Agronomía, Universidad Autónoma de Nuevo León, Francisco Villa S/N, C.P. 66050 General Escobedo, Nuevo León, Mexico; guillermo.ninomd@uanl.edu.mx; 2 Laboratorio de Tecnología de Alimentos, Centro de Investigación en Alimentación y Desarrollo (CIAD) A.C., Coordinación Culiacán, Carretera Culiacán a El Dorado Km 5.5, C.P. 80110 Culiacán, Sinaloa, Mexico; mdmuy@ciad.mx (D.M.-R.); wrubio@ciad.mx (W.R.-C.); perez.briceida@hotmail.com (B.P.-M.); 3 Laboratorio de Química de los Alimentos, Facultad de Salud Públicay Nutrición, Universidad Autónoma de Nuevo León, Av. Dr. Eduardo Aguirre Pequeño y Yuriria, C.P. 64460 Monterrey, Nuevo León, Mexico; ana.dlgarzah@uanl.mx (A.L.d.l.G.); anapatricia.1118@gmail.com (A.P.A.-C.); keelsy.gtz@hotmail.com (K.A.G.-Á.)

**Keywords:** soybean husk, chickpea husk, dietary fiber, white bread, functional properties, nutritional properties

## Abstract

Dietary fiber extracted from soybean and chickpea husks was used in the formulation of white bread. Treatments at different concentrations of dietary fiber (DF): bread + 0.15%, 0.3%, 1.5%, 2% soybean dietary fiber (SDF); bread + 0.15%, 0.3%, 1.5%, 2% chickpea dietary fiber (CDF), and a control treatment (Bread 0% DF) were used initially. However, the treatments that showed the greatest improvement effects were: bread + 2% SDF and bread + 2% CDF. The functionality and the nutritional contribution in the treatments were evaluated during four days of storage. The weight loss on the third day of storage was 30% higher in the control treatment than the products with 2% SDF and 2% CDF, while for the evaluation of firmness, the control obtained a hardness of 86 N, and treatments with 2% SDF and 2% CDF 60 N and 45 N, respectively. The presence of phenolic compounds and their antioxidant activity was evident, mainly in the 2% SDF treatment, which had a total phenolic content of 1036, while in the Bread 0% DF it was 232 mgEAC/kg. The antioxidant activity for 2% SDF by DPPH (2,2-diphenyl-1-picrylhydrazyl), ABTS (3-ethyl-benzothiazoline-6-sulfonic acid), and FRAP (ferric reducing antioxidant power) was 1096, 2567, and 1800 µmolTE/kg, respectively. Dietary fiber addition favored the reduction of weight loss and firmness of white bread during storage. In addition, color was not affected and the content calcium, phenolics, as well as antioxidant capacity were slightly improved.

## 1. Introduction

Current food trends have carried health problems into Mexico: consumption of products rich in carbohydrates and fats but low in fiber favors overweightness and obesity, two risk factors for cardiovascular diseases, diabetes, and others [1,2]. A good source of dietary fiber may come from legumes like soybean (*Glycine max*) and chickpea (*Cicer arietinum*). Up to 40% of their husks contains fiber in the form of celluloses, hemicelluloses, and pectins.

Dietary fiber (DF) is a set of carbohydrates not easily digested by the human body, but its intake carries multiple benefits: it increases the volume of the intestinal bolus, favors intestinal transit, helps prevent colon cancer, and regulates sugar levels in the blood, among other benefits [3]. Dietary fiber is classified as soluble and insoluble. Soluble fiber contains gums, hemicelluloses, mucilages, and pectins, while cellulose and lignins are mostly present in insoluble fiber [4,5].

Phenolic compounds like phenolic acids, tannins, and flavonoids have been reported in the fiber structure as conjugated mono-, di-, and oligosaccharides. These variations have different biological properties, and fiber may be considered a natural source of antioxidants [6,7,8]. The food and agriculture industries produce many phenolic-rich products. Phenolic compounds are secondary plant metabolites produced during growth and reproduction. They play an important protection role against various pathogens and predators. Anti-allergenic, anti-inflammatory, and anti-microbial properties have also been reported for phenolic compounds [9].

This research focuses on alternative uses for underutilized plant materials and waste from the food industry. Great interest exists in usage of industrial food waste that reduces environmental problems and takes advantage of large amounts of biomass for value-added products. Billions of metric tons of biomass are generated from the agricultural industry every year around the world. This waste can be fruit husks, husks from various crops, bagasse, and corn cobs, among others [10]. For example, legume industrialization produces around 400,000 tons of byproducts annually and they are used mainly in animal feed. Such plant materials are an interesting source of nutritional and functional compounds. Many studies have focused on potential uses of these waste materials in the food industry due to their chemical composition, functional properties, and nutraceutical content [11,12].

The objective of this study was to added dietary fiber obtained from soybean and chickpea husks to a white bread formulation and evaluate the changes in its nutritional, functional, and nutraceutical properties.

## 2. Results and Discussion

### 2.1. Weight Loss

In general, weight loss was observed in all of the treatments. However, those added with dietary fiber lost less weight and were statistically different (*p* < 0.05) compared to the control treatment. Table 1 shows the control (Bread 0% DF) treatment had greater weight loss during storage, in terms of water loss of the samples.

The addition of fiber to white bread decreases weight loss proportional to the amount of added fiber in the formulation. This effect was more evident in the 2% treatment on the third day, for both soybean dietary fiber (SDF) and chickpea dietary fiber (CDF), with an approximate weight loss of 30% less compared to the Bread 0% DF. Dietary fiber retains water, which aids in maintaining good texture for longer times [13]. In a study conducted by Bose and Shams [14], chickpea husk was directly used in concentrations of 3% to 5% in cracker formulations based on wheat flour, and this change favored water retention up to 4%.

Other studies have shown the benefits of natural fiber use in the formulation of a wide variety of food products. In the case of bread products, Sivam et al. [15] used 3% soluble dietary fiber (pectin) of a high and low degree of methylation in bread formulations and observed that both types of pectin retained 7% more moisture compared to the control treatment.

### 2.2. Firmness

This texture parameter is one of the most important quality parameters in bread products and represents the force required to deform a product [16]. In Table 2, the effect of storage at room temperature was observed. At day 0, most of the treatments showed no significant difference (*p* > 0.05), except for the treatments 1.5% and 2% SDF that required 31 and 35 N of force, but in general, all the treatments were within the range of 35–40 N. The Bread 0% DF treatment was 300% firmer at day 3 of storage, the SDF and CDF treatments at 0.15 and 0.3% did not show significant differences (*p* < 0.05) compared to the Bread 0% DF treatment during storage, so that treatments did not show a positive effect on the bread. The SDF and CDF treatments at 1.5 and 2% showed a significant effect (*p* > 0.05) from day 1, and on the third day, a difference of 40 N was observed for SDF and CDF at 2% with respect to the Bread 0% DF treatment, a two-fold increase. According to Peighambardoust and Aghamirzaei, [17], the loss of weight and firmness of a baked product are related to loss of moisture and indicate a quickly perishable product. This indicates a physical and chemical deterioration that compromises its texture (hardening) and considerably damages its quality.

Correa et al. [18] reported that adding citrus soluble dietary fiber into the bread formulation decreases the hardness of the bread; the 2% soluble dietary fiber treatment showed 32% lower hardness than the control on the third day of storage. Studies report the use of diverse sources of fibers may have considerable impact on texture, color, and volume characteristics of the final product; however, some results might be contradictory: addition of dietary fiber in the formulation directly increases bread firmness, but in other cases favors softening or development of a loose crumb, depending on the source of pectin used [3,19]. It should be noted that in this study, dietary fiber from soybean husk and chickpea husk had a protective effect during storage of bread products in terms of weight loss. These results agree with Sabanis et al. [20], where they also emphasize addition of dietary fibers in the formulation of various products not only improves their nutritional value but also their shelf life.

According to the results of our study, the addition of dietary fiber at different concentrations has a positive effect on bread products in weight loss and firmness. The effect was more evident at the 2% concentration. Thus, for the next variables, only the results of the bread control and bread treatments with SDF and CDF will be shown at 2%.

### 2.3. Color

Another important parameter for bread quality is color. In the case of white bread (bread roll type), the loaf was characterized by a golden-colored crust and a cream-colored crumb, which together are attractive to the consumer [3]. The acceptance of a food product from consumers depends mainly on its visual aspect. In the case of baking products, and specifically in bread, the color of the crust is an important factor because a good color development of crust is the first aspect evaluated from consumers. Regarding the color evaluation, statistical differences (*p* < 0.05) were observed on day zero of the storage experiment in the a* and b* values, in which the bread control was higher than 2% SDF and 2% CDF, as seen in the view section corresponding to the color parameters of each sample. For the rest of the evaluated days, no significant differences (*p* > 0.05) between the control treatment and the bread treatments with 2% SDF and 2% CDF were observed (Table 3). The information for the color parameters L*, a*, b* for each treatment was generated with ColorHexa software. Our study agrees with the results obtained by Almeida [21], who evaluated the effect of the addition of three different sources of dietary fibers (wheat bran, resistant corn starch, and locust bean gum) to white bread without a high impact on the color parameters. Although there were no statistical differences between treatments in the numerical values of color parameters from day 1 to day 3, the use of L*, a*, b* coordinates in the ColorHexa software revealed the visual color obtained in the crust of the treatments were different in some cases, which is evident for the bread control at day 1, bread 2% CDF at day 2, and bread 2% SDF at day 3.

### 2.4. Dietary Fiber

Table 4 shows a significant difference (*p* < 0.05) in Bread 0% DF treatment with respect to SDF and CDF treatments in content of total dietary fiber, increased by 45% and 39% for bread treatments with 2% SDF and 2% CDF, respectively. Soluble dietary fiber showed increased values of almost 50% in both bread treatments with soybean and chickpea husk dietary fiber compared to the Bread control treatment, while the insoluble dietary fiber did not show significant difference (*p* > 0.05). The soluble dietary fiber consists of hemicelluloses and pectin, and together are approximately 10% of the chickpea husk and up to 10–20% of the soybean husk [22,23].

According to Tosh and Yada [24], the consumption of high-fiber food products has positive effects on human health, such as reducing levels of blood cholesterol and regulating the blood glucose levels, among others. In a study conducted by Vergara et al. [25], mango dietary fiber was used in preparation of biscuits and white bread, and this increased total dietary fiber content with respect to the control treatment, which was similar to the results of our experiment.

### 2.5. Total Phenols and Antioxidant Capacity

Table 5 shows results for the bread control treatment, bread + 2% SDF, and bread + 2% CDF. The effect of the addition of dietary fiber from soybean and chickpea husks can be observed. The Bread 0% DF treatment had 232 mgGAE/kg, while bread + 2% SDF and bread + 2% CDF showed 1036 mgGAE/kg and 1101 mgGAE/kg of phenolics, respectively. The concentration of 2% SDF and 2% CDF treatments was 4.4 and 4.7 times higher than the control treatment, respectively.

The presence of phenolic compounds in bread products is related to their antioxidant activity: the three methods employed in this study showed significant differences between the Bread 0% DF treatment and bread + SDF and CDF treatments. For the DPPH method, the Bread 0% DF treatment reported an antioxidant activity of 353 μmolTE/kg, while products fortified with dietary fiber from soybean and chickpea showed values of 1096 and 1167 μmolTE/kg, respectively. A similar behavior was observed in the ABTS and FRAP methods: 2% SDF and 2% CDF almost doubled their antioxidant activity with respect to the control treatment. For ABTS, the 2% SDF sample showed an increase of 77.6% (2567 μmolTE/kg), while for 2% CDF, it increased by 109% (3035 μmolTE/kg). For FRAP, the antioxidant activity for 2% SDF bread was 2.2 times greater than the Bread 0% DF (818 μmolTE/kg), while for 2% CDF bread, the increase was approximately 1.52 times greater than the control. Bread added with 2% CDF had greater significant differences (*p* < 0.05) than bread added with 2% SDF, with respect to its total fiber content; although both treatments exceeded total fiber content by approximately 4.6 times.

In a previous study by Niño-Medina et al. [26], the researchers evaluated the antioxidant activity of soybean and chickpea husks and reported values in the range of 2350–5280 μmolTE/kg by the DPPH method and 6520–14870 μmolTE/kg by the ABTS method. Several studies report that some sources of dietary fiber from agricultural-derived byproducts have antioxidant properties due to the presence of phenolic compounds. This is the reason why these materials are natural sources of antioxidant compounds in addition to the benefits provided by fiber [27,28].

### 2.6. Minerals

In Table 6, data for the control (Bread 0% DF) treatment, bread + 2% SDF and bread + 2% CDF treatments are shown. No significant differences were observed (*p* > 0.05) for Na, K, and Mg content between treatments; however, an increase of 10% in Ca content in bread added with 2% SDF was significantly different (*p* < 0.05) with respect to the control. Calcium fulfills multiple functions in the organism (growth and development of the human skeleton) and is considered a main supplement along with iron, zinc, magnesium, and potassium, among others; unfortunately, many plant food products lack this mineral [29].

Certain dietary fibers extracted from different plant materials (fruits, cereals or legumes) have various physiological effects in the organism like glucose level regulation, lipid metabolism, and bioavailability of minerals such as calcium and magnesium [30]. For this reason, use or addition of dietary fiber in food products provides health benefits.

## 3. Materials and Methods

### 3.1. Plant Material

Soybean husk, an oil production byproduct, was donated by the company Ragasa Industrias S.A. of C.V., located in Guadalupe, Nuevo León, Mexico. The husks from chickpea were obtained manually from low-quality grains after soaking in water.

### 3.2. Extraction of Dietary Fiber

Dietary fiber extraction was performed according to the method reported by Urías-Orona et al. [23].

### 3.3. Bread Making

Bread products were prepared according to Niño-Medina et al. [31] with modifications. The control was prepared using 1000 g of wheat flour, 620 mL of water, 40 g of sugar, 30 g of yeast, and 20 g of salt. Bread for the treatments also included dietary fiber extracted from soybean husks and chickpea at 0.15%, 0.30%, 1.5%, and 2%. Kneading time was 12 min, followed by a 12 min fermentation at 40 °C, forming pieces of 40 ± 0.5 g, and finally baking at 170 °C for 15 min. After the samples were cooled at room temperature, they were packed in plastic bags and maintained at room temperature for four days (0, 1, 2 and 3).

### 3.4. Weight Loss

The daily weight the pieces of bread per day was recorded during the four days of storage. Samples could cool for 30 min at room temperature, according to the method reported by Demirkensen et al. [32], and weight loss percentage was calculated based on the following formula:

% PP = [(Initial weight (Day 0) − Final weight (Day 1, 2, 3))/Initial weight (Day 0)] × 100

### 3.5. Firmness

Bread firmness was measured with an Ametek Lloyd Instruments Chatillon CS225 (West Sussex, United Kingdom) force tester each storage day. A 40% compression of the average height of the bread pieces was considered according to Jacobs et al. [33], and tests were carried out at a 20 mm/min speed, and using a 5-cm diameter compression plate.

### 3.6. Color

Color determination was carried out using a CR 400 Konica Minolta Chromameter (Tokyo, Japan) for color parameters L*, a*, and b*. For each treatment, three samples were taken: measurements were taken at two points of the bread crust, and the average value of each sample was calculated. Color image was obtained by the ColorHexa software [34] using L*, a*, b* for each sample measurement.

### 3.7. Dietary Fiber

The soluble and insoluble dietary fiber content of bread samples were quantified using Megazyme total dietary fiber kit (Bray, Ireland) according to Dang and Vasanthan [35]. A MES-TRIS (2-(*N*-morpholino)ethanesulfonic acid-tris(hydroxymethyl)aminomethane) buffer (10 mL, 0.05M each, pH 8.2) and thermostable α-amylase (50 μL, 3000 U/mL) were added to the sample (0.3 g) in a 50-mL plastic centrifuge tube with vigorous mixing, and followed by heating the tube in a boiling water bath for 35 min. The tube was cooled down in a 60 °C water bath and rinsed with 15 mL of distilled water. The tube contents were then digested with 100 μL of protease (350 Tyrosine U/mL) for 30 min at 60 °C with continuous agitation and after incubation pH was adjusted to 4.1–4.8 by 0.561 N hydrochloric acid. The tube contents are then further digested with 200 μL of amyloglucosidase (3300 U/mL) for 30 min at 60 °C with continuous agitation. The tube contents were then filtered and washed with distilled water (60 °C) through a celite-in-bed crucible. The residue in the crucible (insoluble dietary fiber) was washed with 95% *v/v* ethanol and dried in an oven (103 °C) overnight. The filtrate and water washes were combined and added with four volumes of preheated (60 °C) 95% *v/v* ethanol to precipitate the soluble dietary fiber for 1 h, filtered and washed with 78% *v/v* ethanol, and then 95% *v/v* ethanol before being left to dry in an oven (103 °C) overnight. Protein and ash contents of dried insoluble dietary fiber and soluble dietary fiber fractions were determined. The insoluble dietary fiber and soluble dietary fiber content was the weight of dried soluble dietary fiber residue minus the weight of protein and ash. The total dietary fiber content was calculated as the sum of insoluble dietary fiber and soluble dietary fiber.

### 3.8. Total Phenols and Antioxidant Capacity

Assays were performed according to López-Contreras et al. [36]. Determination of total phenols (TP) content was carried out using the Folin–Ciocalteu reagent and gallic acid as standard (0 to 200 mg/L). The result was expressed as milligrams of gallic acid equivalents per gram of sample (mgGAE/kg). Antioxidant capacity was evaluated based on the reduction of absorbance of the radicals 2,2-difenyl-1-picrylhydrazilo (DPPH) and 2,2-azino-bis(3-etilbenzotiazolin)-6-sulphonic acid (ABTS), using Trolox as standard (0 to 500 μmol/g), and expressing the results micromoles of Trolox equivalents per gram of sample (μmolTE/g). For the FRAP assay, a working solution was prepared using 300 mM C_2_H_3_NaO_2_-3H_2_O at pH 3.6, 10 mM TPTZ (2,4,6-tripyridyl-striazine) in 40 mM hydrochloric acid, and 20 mM FeCl_3_ 6H_2_O in a 10:1:1 ratio. For the test, 0.2 mL of the phenolic extract was mixed with 3.3 mL of the FRAP reagent. The Trolox standard was used, and the reading was made at 593 nm. Results were expressed as μmolTE/g.

### 3.9. Mineral Content

Mineral content was determined by the Association of Official Analytical Chemist International (AOAC) 955.06 method [37]. Samples were ashed and subjected to acid digestion using HCl. Mineral content was then determined by atomic absorption spectrometry using an Agilent Atomic Absorption 240FS spectrometer (Santa Clara, CA, United States). Potassium (K) and sodium (Na) were analyzed by flame emission at 589.6 and 769.9 nm, respectively, and calcium (Ca), magnesium (Mg), copper (Cu), iron (Fe), and manganese (Mn) were quantified by absorption at 422.7, 285.2, 213.9, 324.7, 248.3, and 279.5 nm, respectively.

### 3.10. Statistical Analysis

All tests were performed in triplicate. Data analysis was performed by one-way analysis of variance (ANOVA) for each of the tested variables in Minitab software. The mean difference was analyzed with the Tukey test at 95% significance level (*p* < 0.05).

## 4. Conclusions

In this study, we observed the positive effect of added dietary fiber from chickpea and soybean husks into the formulation of white bread. Dietary fiber addition favored the reduction of weight loss and firmness during storage. Color, an important quality parameter, was not affected compared to the bread control. Calcium content was slightly improved, as well as antioxidant activity and phenolic compounds content. There is limited literature available for comparison with the current report. However, from the results obtained we concluded that an improvement effect in shelf life and rheological, physical, and sensory parameters was observed. It is suggested that the possible health benefits of the dietary fiber addition to baking products will be study in the future.

## Figures and Tables

**Table 1:** Weight loss in bread products during storage at room temperature.

Treatment	Weight Loss (%)		
	Day 1	Day 2	Day 3
Bread Control (0% DF)	0.45 ± 0.01 ^a^	0.91 ± 0.01 ^a^	1.38 ± 0.01 ^a^
Bread + 0.15% SDF	0.43 ± 0.01 ^a,b^	0.79 ± 0.01 ^b^	1.19 ± 0.01 ^b,c^
Bread + 0.15% CDF	0.48 ± 0.01 ^a^	0.84 ± 0.02 ^a,b^	1.20 ± 0.03 ^a,b^
Bread + 0.3% SDF	0.37 ± 0.01 ^b,c,d^	0.69 ± 0.01 ^c^	1.09 ± 0.03 ^c,d^
Bread + 0.3% CDF	0.41 ± 0.01 ^a,b,c^	0.82 ± 0.01 ^b^	1.18 ± 0.02 ^b,c^
Bread + 1.5% SDF	0.29 ± 0.03 ^e^	0.72 ± 0.01 ^c^	1.12 ± 0.03 ^b,c^
Bread + 1.5% CDF	0.30 ± 0.02 ^d,e^	0.70 ± 0.01 ^c^	1.06 ± 0.01 ^c,d^
Bread + 2% SDF	0.32 ± 0.01 ^d,e^	0.37 ± 0.01 ^e^	0.77 ± 0.08 ^e^
Bread + 2% CDF	0.35 ± 0.02 ^c,d,e^	0.58 ± 0.02 ^d^	0.94 ± 0.01 ^d^

Different letters within the same column are significantly different (*n* = 3). DF = Dietary Fiber, SDF = Soybean Dietary Fiber, CDF = Chickpea Dietary Fiber.

**Table 2:** Firmness in bread products during storage at room temperature.

Treatment	Firmness (N)			
	Day 0	Day 1	Day 2	Day 3
Bread Control (0% DF)	39.0 ± 1.3 ^a^	76.9 ± 1.1 ^a^	114.5 ± 0.7 ^a^	125.4 ± 2.7 ^a^
Bread + 0.15% SDF	39.1 ± 1.5 ^a^	77.0 ± 0.2 ^a^	114.4 ± 1.3 ^a^	125.4 ± 2.7 ^a^
Bread + 0.15% CDF	39.0 ± 0.3 ^a^	76.0 ± 0.2 ^a^	113.4 ± 1.9 ^a^	126.3 ± 2.2 ^a^
Bread + 0.3% SDF	38.0 ± 3.4 ^a,b^	75.6 ± 1.7 ^a^	114.0 ± 1.4 ^a^	125.0 ± 1.4 ^a^
Bread + 0.3% CDF	38.0 ± 1.2 ^a^	76.0 ± 1.2 ^a^	113.9 ± 1.3 ^a^	124.0 ± 2.8 ^a^
Bread + 1.5% SDF	31.1 ± 2.8 ^b^	69.5 ± 1.6 ^b^	100.3 ± 2.0 ^b^	109.0 ± 2.0 ^b^
Bread + 1.5% CDF	35.8 ± 2.0 ^a,b^	73.9 ± 1.3 ^a,b^	96.7 ± 0.2 ^b^	106.4 ± 3.0 ^b^
Bread + 2% SDF	35.8 ± 1.0 ^b^	57.5 ± 1.6 ^c^	63.9 ± 0.3 ^c^	86.0 ± 3.5 ^c^
Bread + 2% CDF	38.8 ± 0.5 ^a^	52.9 ± 0.4 ^c^	65.9 ± 1.5 ^c^	84.4 ± 0.8 ^c^

Different letters within the same column are significantly different (*n* = 3). DF = Dietary Fiber, SDF = Soybean Dietary Fiber, CDF = Chickpea Dietary Fiber.

**Table 3:** Chromatic characteristics for bread products during storage at room temperature.

Storage Period	Treatment	Chromatic Parameter			
		L*	a*	b*	Color View
Day 0	Bread Control (0% DF)	64.2 ± 0.5 ^a^	13.4 ± 0.2 ^a^	32.9 ± 0.3 ^a^	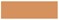
	Bread + 2% SDF	67.7 ± 4.0 ^a^	11.9 ± 1.7 ^a,b^	28.6 ± 1.9 ^b^	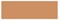
	Bread + 2% CDF	70.8 ± 2.3 ^a^	9.2 ± 0.9 ^b^	27.1 ± 0.2 ^b^	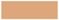
Day 1	Bread Control (0% DF)	67.0 ± 6.0 ^a^	10.9 ± 2.9 ^a^	29.0 ± 1.8 ^a^	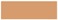
	Bread + 2% SDF	72.8 ± 0.2 ^a^	7.1 ± 1.0 ^a^	25.7 ± 1.6 ^a^	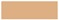
	Bread + 2% CDF	70.8 ± 6.7 ^a^	8.5 ± 5.0 ^a^	26.8 ± 4.0 ^a^	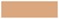
Day 2	Bread Control (0% DF)	66.4 ± 3.0 ^a^	10.6 ± 2.2 ^a^	27.2± 4.3 ^a^	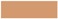
	Bread + 2% SDF	67.2 ± 0.2 ^a^	10.2 ± 0.2 ^a^	39.8 ± 3.2 ^a^	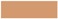
	Bread + 2% CDF	72.1 ± 1.8 ^a^	8.1 ± 1.8 ^a^	27.8 ± 0.4 ^a^	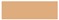
Day 3	Bread Control (0% DF)	63.6 ± 0.9 ^a^	11.7 ± 1.2 ^a^	29.5 ± 2.0 ^a^	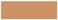
	Bread + 2% SDF	60.9 ± 5.8 ^a^	12.1 ± 2.2 ^a^	29.9 ± 0.7 ^a^	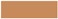
	Bread + 2% CDF	66.3 ± 6.2 ^a^	9.8 ± 3.2 ^a^	28.7 ± 1.5 ^a^	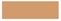

Different letters within the same column in the same day of the evaluation are significantly different (*n* = 3). DF = Dietary Fiber. SDF = Soybean Dietary Fiber, CDF = Chickpea Dietary Fiber.

**Table 4:** Dietary fiber content in bread products at day 0.

Treatment	Dietary Fiber (%)		
	Insoluble	Soluble	Total
Bread Control (0% DF)	2.3 ± 0.2 ^a^	2.6 ± 0.1 ^b^	4.9 ± 0.3 ^b^
Bread + 2% SDF	3.1 ± 0.1 ^a^	4.1 ± 0.3 ^a^	7.1 ± 0.3 ^a^
Bread + 2% CDF	2.7 ± 0.3 ^a^	4.2 ± 0.2 ^a^	6.9 ± 0.6 ^a^

Different letters within the same column are significantly different (*n* = 3). DF = Dietary Fiber, SDF = Soybean Dietary Fiber, CDF = Chickpea Dietary Fiber.

**Table 5:** Total phenols content and antioxidant capacity in bread products at day 0.

Treatment	Total Phenols (mgGAE/kg)	Antioxidant Capacity (μmolTE/kg)		
		DPPH	ABTS	FRAP
Bread Control (0% DF)	232 ± 29 ^c^	354 ± 40 ^b^	1445 ± 146 ^b^	819 ± 72 ^c^
Bread + 2% SDF	1036 ± 5 ^b^	1097 ± 36 ^a^	2567 ± 94 ^a^	1800 ± 5 ^b^
Bread + 2% CDF	1102 ± 6 ^a^	1168 ± 88 ^a^	3025 ± 626 ^a^	1247 ± 29 ^a^

Different letters within the same column are significantly different (*n* = 3). DF = Dietary Fiber, SDF = Soybean Dietary Fiber, CDF = Chickpea Dietary Fiber, mgGAE/kg = milligrams of gallic acid equivalents per kilogram, μmolTE/kg = micromoles trolox equivalents per kilogram.

**Table 6:** Mineral content in bread products at day 0.

Treatment	Minerals (mg/100 g)			
	Na	K	Ca	Mg
Bread Control (0% DF)	9032 ± 141 ^a^	1995 ± 21 ^a^	1956 ± 28 ^b^	393 ± 4 ^a^
Bread + 2% SDF	9747 ± 46 ^a^	2017 ± 93 ^a^	2154 ± 30 ^a^	417 ± 3 ^a^
Bread + 2% CDF	9684 ± 317 ^a^	2059 ± 36 ^a^	1991 ± 32 ^b^	391 ± 9 ^a^

Different letters within the same column are significantly different (*n* = 3). DF = Dietary Fiber, SDF = Soybean Dietary Fiber, CDF = Chickpea Dietary Fiber.
